# Editorial: The (un)desirability of cell death in health and disease: emerging modulators and their mechanisms

**DOI:** 10.3389/fcell.2024.1523293

**Published:** 2024-11-19

**Authors:** Daniel José Barbosa, Joana Barbosa, Elaine M. Souza-Fagundes, Patrícia M. A. Silva

**Affiliations:** ^1^ Associate Laboratory i4HB - Institute for Health and Bioeconomy, University Institute of Health Sciences - CESPU, Gandra, Portugal; ^2^ UCIBIO – Applied Molecular Biosciences Unit, Translational Toxicology Research Laboratory, University Institute of Health Sciences (1H-TOXRUN, IUCS-CESPU), Gandra, Portugal; ^3^ I3S - Instituto de Investigação e Inovação em Saúde, Universidade do Porto, Porto, Portugal; ^4^ Departamento de Fisiologia e Biofísica, Universidade Federal de Minas Gerais, Belo Horizonte, Minas Gerais, Brazil; ^5^ UNIPRO – Oral Pathology and Rehabilitation Research Unit, University Institute of Health Sciences (IUCS-CESPU), Gandra, Portugal

**Keywords:** cell death modulators, apoptosis, ferroptosis, autophagy, cancer, neurodegenerative diseases, cell death mechanisms

**FIGURE 1:**
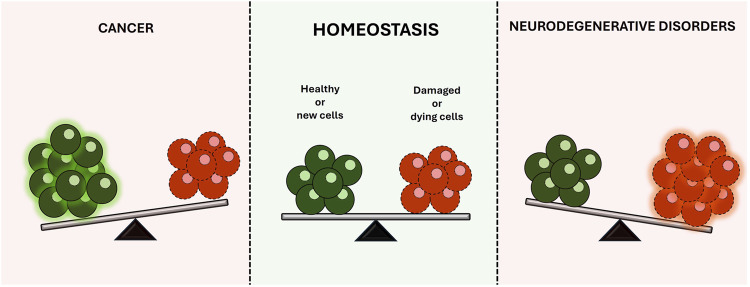
Balanced regulation of cell death and survival, illustrating how disruptions can lead to disease development.

The balanced regulation of cell death and survival is critical for maintaining the homeostasis of the human body. Disruption of this delicate equilibrium often underlies the development of several diseases. For instance, cancer cells often develop resistance to natural cell death mechanisms, leading to uncontrolled proliferation and malignant growth (Maleki et al., 2023; Fatma and Siddique, 2024). Conversely, neurodegenerative diseases involve the progressive loss of specific brain cell populations, resulting in a broad spectrum of debilitating symptoms and, ultimately, cell death (Nixon, 2024; Baev et al., 2024) (Figure 1).

The incidence and prevalence of both cancer and neurodegenerative diseases are rising rapidly, significantly impacting patients’ quality of life and creating major economic and healthcare challenges worldwide (Muralidharan et al., 2023; Dorsey et al., 2013). While knowledge about the role of abnormal cell death in these diseases has advanced, the precise mechanisms driving these processes, as well as effective strategies to modulate them, remain only partially understood. This limited understanding complicates efforts to tailor treatments to different disease types. For instance, in cancer, therapeutic approaches focus on inducing cell death to eliminate malignant cells, whereas in neurodegenerative diseases, the goal shifts to preventing cell death to preserve affected neurons and delay symptom progression.

This Research Topic focused on enriching our understanding of the emerging modulators of cell death, exploring their mechanisms of action and potential therapeutic applications in various disease scenarios.

In total, two original research articles and three review articles were published. This editorial briefly summarizes the findings and highlights key insights derived from these articles, emphasizing their contributions to the field and the potential they present for guiding future research and/or therapeutic strategies.

All the published studies converge to explore the intricate roles of cell death mechanisms across different contexts.

The exploration of cellular responses to different agents provides valuable insights into the mechanisms of cell death and the potential for therapeutic interventions. In this context, Mallardo et al. investigated the effects of dimethyl sulfoxide (DMSO), commonly used as a solvent in pharmacological studies, on neuronal and glial cell viability, as well as the potential neuroprotective role of lignanamides (LnHS) in counteracting DMSO-induced cytotoxicity. Using SH-SY5Y and U-87 cell lines as *in vitro* models for neurons and glia, respectively, the authors assessed cell viability, proliferation, autophagy, oxidative stress, and inflammatory responses after treatment with DMSO and/or LnHS. They showed that DMSO decreases cell viability in both neuronal and glial cells, whereas LnHS does not impair SH-SY5Y cell viability. Co-administration of LnHS with DMSO partially restored SH-SY5Y cell survival and colony-forming capacity, while reducing oxidative stress markers, such as nitrite release and reactive oxygen species (ROS) generation, and modulating inflammatory responses, notably increasing interleukin 8 (IL-8) and counteracting DMSO-induced interleukin 6 (IL-6) production. These results suggest that LnHS may serve as a promising neuroprotective agent, counteracting the cytotoxic effects of DMSO and supporting neuronal health. Further studies exploring the molecular mechanisms underlying LnHS activity could provide valuable insights into its biological effects.

Kamran et al. delved into the mechanism by which D,L-methadone induces apoptosis in acute lymphoblastic leukemia (ALL) cells, a type of hematologic cancer primarily affecting children. Previous research demonstrated that D,L-methadone triggers apoptosis through μ-opioid receptor 1 (OPRM1)-mediated endoplasmic reticulum (ER) calcium release (Lee et al., 2021). The results of this study revealed that the D,L-methadone-induced ER Ca^2+^ release in ALL cells is dependent on the G_αi_ subunit, as its inhibition blocks this process without affecting G_βϒ_. Moreover, further experiments showed that activating adenylyl cyclase (AC) with forskolin or using 8-CPT-cAMP inhibits D,L-methadone-induced ER Ca^2+^ release, suggesting a G_αi_-mediated downregulation of AC and cAMP. Notably, the protein kinase A (PKA) inhibitor 14–22 amide (myr) can independently elicit ER Ca^2+^ release, indicating that PKA inhibition is a crucial step in the D,L-methadone-induced ER Ca^2+^ release mechanism. This process is linked to decreased phosphorylation of phospholipase C β (PLCβ3) and BCL2 associated agonist of cell death (BAD), ultimately leading to caspase activation and apoptosis. Globally, the findings highlight that D,L-methadone induces ER Ca^2+^ release and apoptosis in ALL cells through a G_αi_-dependent pathway involving the downregulation of AC-cAMP-PKA-PLCβ3/BAD signaling. The ability of the PKA inhibitor alone to kill ALL cells suggests that targeting PKA could be a promising therapeutic strategy for treating ALL.

In a broader scope, Han et al. dissected the role of noncoding RNAs in programmed cell death related to osteoporosis, illustrating how these molecules influence cell death mechanisms and contribute to bone homeostasis disruption. Importantly, the authors highlight the role played by the noncoding RNA-mediated programmed cell death pathway in the genetic regulation associated with osteoporosis. Globally, this review pointed out a unique opportunity to develop innovative diagnostic and therapeutic strategies for osteoporosis.

Expanding the discussion of cellular degradation processes, Zhou et al. provided an overview of macroautophagy’s protective role in pancreatic health. The authors connected autophagy to various pancreatic diseases, detailing how disruptions in this pathway can lead to oxidative stress, inflammation, and cell death. This elegant review reinforces the theme of balancing cell death and survival, revealing how autophagy manipulation may serve as a therapeutic avenue, akin to the strategies discussed in the other articles.

Meng et al. addressed the pathogenicity of *Salmonella,* a prevalent foodborne pathogen that affects both humans and animals, and its link to pyroptosis, a pro-inflammatory form of programmed cell death that plays a crucial role in maintaining intestinal homeostasis. Their insights into the virulence factors of *Salmonella* and the mechanisms behind pyroptosis further contribute to our understanding of cell death in infectious diseases. This emphasizes the need for continued research to uncover regulatory mechanisms and develop clinical strategies for the prevention and treatment of *Salmonella* infections and related diseases.

Together, these contributions form a cohesive narrative around the desirability and modulation of cell death, shedding light on potential therapeutic interventions for various health conditions. The intersection of basic research and clinical application drives the ongoing effort to discover novel regulators that can effectively modulate these pathways, ultimately enhancing patient outcomes and advancing the field of biomedical science.
